# The effects of tramadol administration on hippocampal cell apoptosis, learning and memory in adult rats and neuroprotective effects of crocin

**DOI:** 10.1007/s11011-018-0194-6

**Published:** 2018-02-22

**Authors:** Farideh Baghishani, Abbas Mohammadipour, Hossain Hosseinzadeh, Mahmoud Hosseini, Alireza Ebrahimzadeh-bideskan

**Affiliations:** 1 Department of Anatomy and Cell Biology, School of Medicine, Mashhad University of Medical Sciences, Azadi Sq., Vakilabad Blvd, P.O. Box 91779-48564, Mashhad, Iran; 2 Microanatomy Research Center, School of Medicine, Mashhad University of Medical Sciences, Mashhad, Iran; 3 Pharmaceutical Research Center, Department of Pharmacodynamics and Toxicology, School of Pharmacy, Mashhad University of Medical Sciences, Mashhad, Iran; 4 Division of Neurocognitive Sciences, Psychiatry and Behavioral Sciences Research Center, Mashhad University of Medical Sciences, Mashhad, Iran

**Keywords:** Tramadol, Crocin, Apoptosis, Memory, Morris water maze, Passive avoidance

## Abstract

Tramadol, a frequently used pain reliever drug, present neurotoxic effects associated to cognitive dysfunction. Moreover, crocin has been reported to have neuroprotective effects. The aim of this study was to assess crocin’s capacity to protect learning, and memory abilities on tramadol-treated rats. A total of 35 rats were divided into five groups: Control, Saline, tramadol (50 mg/kg), tramadol + crocin(30 mg/kg), crocin groups and treated orally for 28 consecutive days. Morris water maze (MWM) and passive avoidance (PA) tests were done, followed by dissection of the rat’s brains for toluidine blue and TUNEL staining. In MWM test, tramadol group spent lower time and traveled shorter distance in the target quadrant (Q1) *(P < 0.05)*. On the other side, the traveled distance in tramadol-crocin group was higher than tramadol *(P < 0.05)*. In PA test, both the delay for entering the dark, and the total time spent in the light compartment decreased in tramadol comparing to the control group *(P < 0.05)*, while it increased in tramadol-crocin compared with the tramadol group *(P < 0.05)*. In tramadol-treated animals, the dark neurons (DNs) and apoptotic cells in CA1, CA3 and DG increased *(P < 0.05)*, while concurrent intake of crocin decreased the number of DNs and apoptotic cells in these areas *(P < 0.05)*. Crocin was able to improve learning and memory of tramadol-treated rats and also decreased DNs and apoptotic cells in the hippocampus. Considering these results, the potential capacity of crocin for decreasing side effects of tramadol on the nervous system is suggested.

## Introduction

Tramadol is a synthetically produced opioid with a specific chemical formula; C16- H25- NO2 (Carrillo-Munguía et al. 2015; Shah et al. 2013; Matthiesen et al. 1998). Tramadol is an analgesic drug with a mechanism of action influencing pain sensation pathways (Mohamed et al. 2015; Shah et al. 2013). This drug is used for the prevention and treatment of moderate to severe pains (Abdel-Zaher et al. 2011; Elkhateeb et al. 2015; Matthiesen et al. 1998). Also, it is an analgesic of choice during and the postoperative period, with a dose adjusted to the severity of the patient’s pain and sensitivity (Elkhateeb et al. 2015; Matthiesen et al. 1998). Nowadays, this opioid drug has become one of the most widely prescribed drugs in the world (Zhuo et al. 2012). However, studies have shown that long-term use of the opioids is associated with addiction physical and psychological dependence (Ghoneim et al. 2014). Despite its therapeutic effects, tramadol also has harmful effects on various organs. This opioid drug has hepatotoxicity and nephrotoxicity effects and can cause liver and kidney damages (Elkhateeb et al. 2015). Tramadol is a lipophilic opioid which, due to freely crossing the placenta, causes withdrawal syndrome in neonatal (De Wit and Koomen-Botman 2012). The effects of its neurotoxicity have been proven and continuous administration of tramadol resulted in weight loss of rats’ brain (Zhuo et al. 2012). Some of the new studies have also demonstrated that tramadol administration impairs memory function in rodent models by activation of μ-opioid receptors (Hosseini-Sharifabad et al. 2016; Yan et al. 2011). On the other hand, chronic administration of tramadol cause histological abnormalities such as increasing apoptosis in rat cerebral cortex associated with oxidative stress (Ghoneim et al. 2014). Other studies have illustrated that tramadol can induce seizures in patients (Boostani and Derakhshan 2012). Additionally, recent studies have shown that tramadol leads to increasing oxidative stress in various tissues such as the brain (Abdel-Zaher et al. 2011). Tramadol has also been observed to decrease the expression of some genes in the hippocampus (Candeletti et al. 2006).

The hippocampus is a part of the limbic system and one of the most important areas of the brain due to its role in learning and spatial memory (Bagheri-abassi et al. 2015; Daulatzai 2013; Mohammadipour et al. 2016; Tamaddonfard et al. 2013). The hippocampus high metabolic activity makes it very vulnerable to oxidative stress (Cui et al. 2004; Mohammadipour et al. 2016). Furthermore, it is believed that dark neurons (DNs) and apoptotic neurons, demonstrate a common way of neuronal cell death with some differences in intracellular pathways (Ahmadpour and Haghir 2011). In rat hippocampus, DNs can be detected by toluidine blue staining, and apoptosis cells can be recognized by TUNEL staining (Bagheri-abassi et al. 2015).

Saffron or *Crocus sativus* L. is a perennial herb of the family Iridaceae (Ghasemi et al. 2015). One of the active ingredients of saffron is crocin, which is a water-soluble carotenoid (Ghadrdoost et al. 2011; Hassani et al. 2014). Saffron is cultivated in Iran and is widely used in traditional and modern medicine (Ghasemi et al. 2015; Hassani et al. 2014). Crocin has antioxidant properties, and its protective effects have been proven on various organs (Chahine et al. 2013; Hassani et al. 2014; Samarghandian et al. 2014). It has been demonstrated that crocin can act as a neuroprotectant (Purushothuman et al. 2013; Sadeghnia et al. 2013). It also specified that crocin can increase levels of some neuropeptides and neurotrophic factors such as nerve growth factor inducible (VGF) and brain-derived neurotrophic factor (BDNF) in rat’s hippocampus (Ghasemi et al. 2015; Hassani et al. 2014).

So far, there are some studies related to neurotoxic effects of tramadol on the brain and also learning and memory impairments, but there is no document about learning mechanism and memory impairment after tramadol treatment. Moreover, the neuroprotective effects of crocin on the hippocampal tissues, learning and memory abilities in tramadol-treated rats has not been studied yet. So, in this study, tramadol neurotoxic effects and neuroprotective impacts of crocin in the adult rat’s hippocampus was studied.

## Materials and methods

### Drugs and preparation

The tablets of tramadol used in this study were purchased from Modava, Co. (Iran). Crocin used was also purchased from Bu Ali Research Institute. (Mashhad, Iran). Drugs were suspended in normal saline solution and freshly administered to the rats by feeding them through needle.

### Animals and treatment

In this study, 35 adult male Wistar rats weighting in 220–250 g were prepared from the Animal House of Mashhad University of Medical Sciences. The rats were kept in standard cages, under controlled conditions of temperature (20–22 °C) and lighting (12 h light/dark). Animals in this study were permitted free access to food and water.

### Experimental procedure

After adaptation, the rats were randomly divided into five groups (*N = 7*); tramadol group: the animals receiving 50 mg/kg tramadol (Abdel-Zaher et al. 2011; Ghoneim et al. 2014), tramadol-crocin group: the animals receiving 50 mg/kg tramadol and 30 mg/kg crocin, crocin group: the animals receiving 30 mg/kg crocin (Bandegi et al. 2014; Tamaddonfard et al. 2013), saline group: the animals receiving normal saline, control group: without any intervention. All the administrations were daily done over for 28 consecutive days.

All related experimental procedures were approved by Animal Ethic Committee of Mashhad University Medical Sciences on Animal Research.

### Behavioral tests

#### Morris water maze test

Morris water maze (MWM) composed of a black circular pool (136 cm diameter, 60 cm high and 30 cm depth) and filled with water (23–25 °C) was set up in the center of a small room. A circular platform (10 cm diameter and 28 cm high) was located into the pool and was submerged about 2 cm beneath the water surface in the center of the northeast quadrant. Outside the MWM, some steady visual cues were available in various situations around the room, for example, computer, hardware, and posters. The rats performed four trials in each of the five consecutive days. Each trial began with the rat being placed in the pool and released facing the sidewall at one of the four locations. The boundaries of the four quadrants and apparatus were divided into four quadrants (North (N), East (E), South (S), and West (W). The released locations were randomly predetermined. For every trial, the animal was allowed to swim until it found and remained on the platform for 20 s. If 60 s passed and the rat didn’t find the platform, it would be guided to the platform by the experimenter. Then, the rat was allowed to stay there for 20 s. At the end of four trials, the rat was removed from the pool, dried, and placed in its holding bin for 20 s. The time spent and the traveled distance to reach the platform was recorded by a video tracking system. On the sixth day, the platform was removed. Then, the rat was allowed to swim for 60s and the time spent and traveled distance in the target quadrant (Q1) were compared among the groups (Huang et al. 2016; Mohammadipour et al. 2016).

#### Passive avoidance test

The passive avoidance (PA) apparatus consisted of a light and a dark compartment with the same size (20 × 20 × 30 cm) separated by a small gate. The floor of the two light and dark compartments were made of stainless-steel bars (0.5 cm diameter) separated by a distance of 1 cm. The rats were accustomed to the PA apparatus during two consecutive days (5 min in each day) before the training or testing sessions. All training or testing sessions were carried out between 8:00 and 11:30 a.m. On the third day, every animal was placed in the light compartment for 20 s. After the small door was opened, the time delay for entering the dark compartment was recorded. Also, the total time spent in the two light and dark compartments were recorded, and each rat was removed from the experiment when it waited for more than 180 s to cross the other side. In the training session, the rat was placed in the light compartment facing away from the dark compartment, and when the rat entered completely into the dark compartment, the door was closed, and it received an electric shock (50 Hz, 3 s, and 1 mA intensity). Then, the rat was returned to its home cage. Three, 24, and 48 h after training to do retention phase or test phase, the animal was placed in the light compartment and the time delay for entering the dark compartment as well as the time spent by the animal in the dark and light compartments was recorded and defined as the retention trial (Manral et al. 2016; Mohammadipour et al. 2016).

### Histological methods

#### Tissue sampling

After completing MWM and PA tests, and being anesthetized by chloroform, all the rats were sacrificed. Then the brains were collected carefully and washed with normal saline. The brains of each group were fixed with 10% normalin for five days.

#### Toluidine blue staining

After fixation, the tissue specimens were dehydrated, cleared with xylene and embedded in paraffin. The brain tissue blocks were cut into 5 μm coronal serial sections (5 sections in each animal at 100 μm intervals) and stained with toluidine blue (Bagheri-abassi et al. 2015; Sadeghi et al. 2013).

#### Apoptotic cell death staining

The apoptotic cells identification was performed using the TUNEL technique. Coronal serial sections with 5 μm thickness were used for TUNEL method. Then tissue sections were deparaffinized with xylene, rehydrated through descending concentrations of ethanol and rinsed in 0.1 M phosphate buffered saline (PBS) twice for 10 min at room temperature. In order to chromatin protein digestion, 20 μg/ml proteinase-K was applied for 20 min at room temperature. Then after washing in PBS, TUNEL reaction was performed using the cell death detection kit (Roche, Germany) based on our previous study protocol. In this method, the nuclei of apoptotic cells were identified by the presence of dark brown staining (Ataei and Ebrahimzadeh-bideskan 2014; Bagheri-abassi et al. 2015).

### Measurements

Different regions of the hippocampus were photographed using the microscope (*Olympus BX51, Japan*) with a × 40 objective lens (*UPlan FI, Japan*). The number of DNs and apoptotic cells were counted under 40 × objectives then by using rectangular grids placed randomly in the investigated areas. Morphometrical methods were applied to count DNs and apoptotic cells per unit area in CA1, CA2, CA3 and dentate gyrus (DG) subdivisions of the hippocampus. Finally, the mean numbers of DNs and apoptotic cells per unit area (NA) in different regions of the hippocampus were calculated using the following formula (Bagheri-abassi et al. 2015; Sadeghi et al. 2013).

$$ {\mathrm{N}}_{\mathrm{A}}=\frac{\sum \overline{\mathrm{Q}}}{\mathrm{a}/\mathrm{f}.\sum \mathrm{P}} $$

In this formula, “*ΣQ*“ is the sum of counted particles appeared in sections, “*a/f*” is the area associated with each frame and “*ΣP*” is the sum of the frame associated points hitting the specified space.

### Statistical analysis

Statistical analysis was performed using the SPSS 16 software for windows and the acquired data from the DNs and apoptotic cells counting methods were reported as mean ± SEM. Then, the data was analyzed by using one-way analysis of variance (ANOVA) & Tukey statistical tests. *P < 0.05* was considered to be statistically significant.

## Results

### Morris water maze

**Fig. 1:**
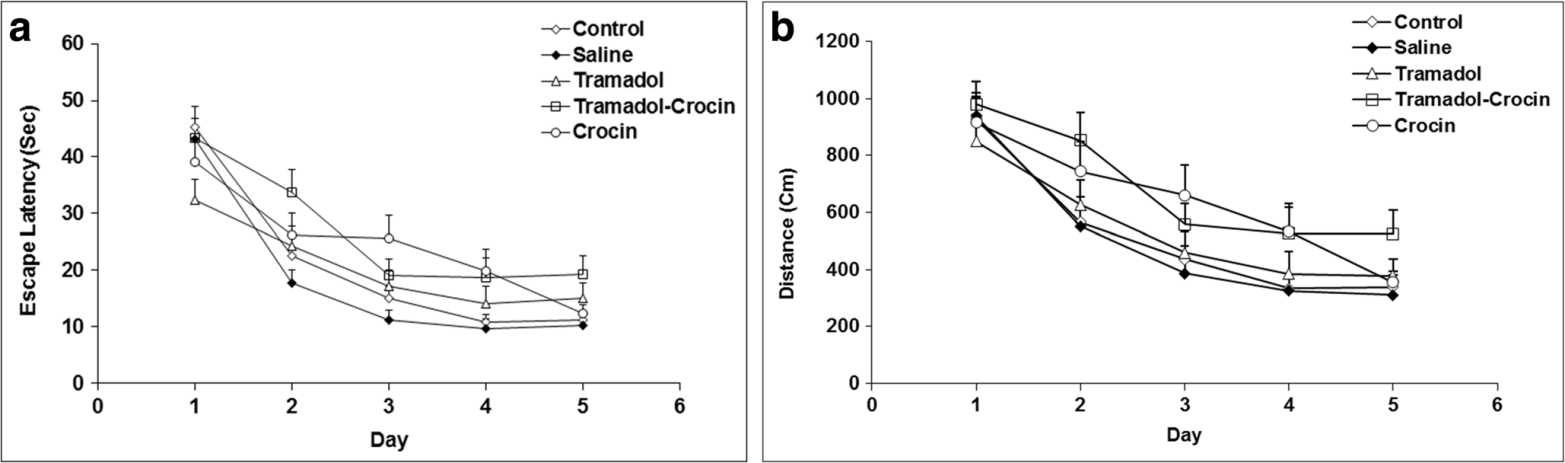
Comparison of (**a**) time spent and (**b**) the traveled distance in the MWM test between the five groups using repeated measures ANOVA. Data is expressed as mean ± SEM (*n = 7* in each group)

In the MWM test, the results showed that there was no significant difference either in the time spent or in the traveled distance to reach the platform between the five groups during 5 days (Fig. 1a and b).

**Fig. 2:**
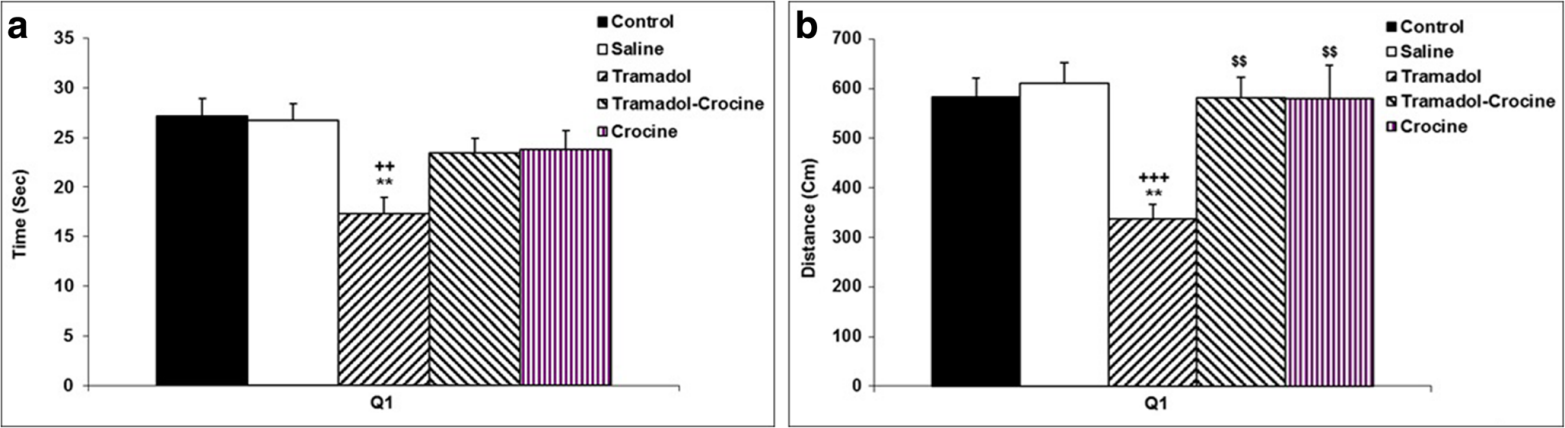
Comparison of (**a**) time spent and (**b**) the traveled distance in the MWM test in the target quadrant (Q1) and other quadrants between the five groups using repeated measures ANOVA. Data are expressed as mean ± SEM (*n = 7* in each group). ^**^ *P < 0.01* Tramadol group compared with Control group, ^++^ *P < 0.01* (a), ^+++^ *P < 0.001* (b) Tramadol group compared with Saline group, ^$$^ *P < 0.01* Crocin and Tramadol-Crocin groups compared with Tramadol group

In the probe day, the time spent and the traveled distance in the target quadrant (Q1) were significantly lower in the tramadol group in comparison with the control and saline groups (*P < 0.01* - *P < 0.001*) (Fig. 2a and b). Also, the amount of the time spent in the target quadrant (Q1) showed that there was no difference between the animals of crocin and tramadol-crocin groups and the tramadol group (Fig. 2a), while the traveled distance in crocin and tramadol-crocin groups was significantly higher than the tramadol group (*P < 0.001*) (Fig. 2b). Furthermore, there was no significant difference in the time spent and the traveled distance in the target quadrant (Q1) between crocin and tramadol-crocin groups (Fig. 2a and b).

### Passive avoidance

**Fig. 3:**
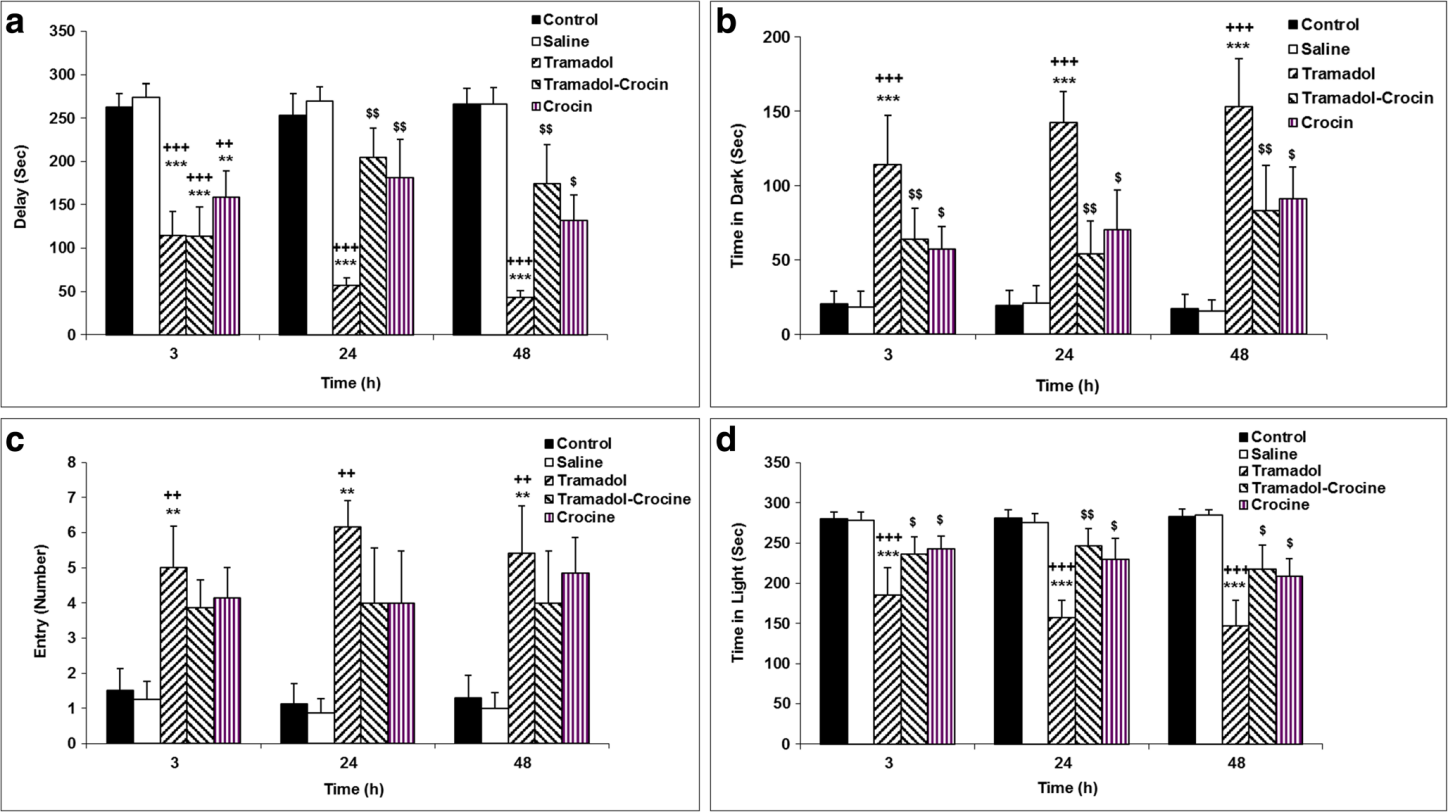
(**a**) Comparison of time delay for entering the dark compartment in the PA test at 3, 24 and 48 h after receiving the shock between the five groups using repeated measures ANOVA. Data is expressed as mean ± SEM (*n = 7* in each group). ^**^ *P < 0.01*, ^***^ *P < 0.001* Crocin, Tramadol-Crocin and Tramadol groups compared with the Control group, ^++^ *P < 0.01*, ^+++^ *P < 0.001* Crocin, Tramadol-Crocin and Tramadol groups compared with Saline group, ^$^ *P < 0.05*, ^$$^ *P < 0.01* Crocin and Tramadol-Crocin groups compared with Tramadol group **(b)** Comparison of total time spent in the dark compartment in the PA test at 3, 24 and 48 h after receiving the shock between the five groups using repeated measures ANOVA. Data are expressed as mean ± SEM (*n = 7* in each group). ^***^ *P < 0.001* Tramadol group compared with Control group, ^+++^ *P < 0.001* Tramadol group compared with Saline group, ^$^ *P < 0.05*, ^$$^ *P < 0.01* Crocin and Tramadol-Crocin groups compared with Tramadol group **(c)** Comparison of the number of entries to the dark compartment in the PA test at 3, 24 and 48 h after receiving the shock between the five groups using repeated measures ANOVA. Data are expressed as mean ± SEM (*n = 7* in each group). ^**^ *P < 0.01* Tramadol group compared with Control group, ^++^ *P < 0.01* Tramadol group compared with Saline group **(d)** Comparison of total time spent in the light compartment in the PA test at 3, 24 and 48 h after receiving the shock between the five groups using repeated measures ANOVA. Data are expressed as mean ± SEM (*n = 7* in each group). ^***^ *P < 0.001* Tramadol group compared with Control group, ^+++^ *P < 0.001* Tramadol group compared with Saline group, ^$^ *P < 0.05*, ^$$^ *P < 0.01* Crocin and

Three, twenty four and forty eight hours after receiving electric shock, the results of PA test showed that the time delay for entering the dark compartment in the tramadol group was significantly lower in comparison to the control and saline groups (*P < 0.001*). The results also showed that three hours after receiving the shock, the time delay for entering the dark compartment in the tramadol-crocin group was lower than the control and saline groups (*P < 0.001*), while 24 and 48 h after receiving the shock, the time delay for entering the dark compartment in the tramadol-crocin group was higher than the tramadol group (*P < 0.01*). Additionally, time delay by the animals of crocin group was lower than the control and saline groups at 3 h after receiving the shock (*P < 0.01*). Furthermore, the animals of crocin group increased the time delay for entering the dark compartment 24 and 48 h after receiving a shock in comparison with tramadol group (*P < 0.05* - *P < 0.01*), and there was no significant difference in the time delay for entering the dark compartment in crocin and tramadol-crocin groups (Fig. 3a).

The results of this test also showed that 3, 24 and 48 h after receiving the shock, the total time spent in the dark compartment in the tramadol group was significantly higher than the control and saline groups (*P < 0.001*). 3, 24 and 48 h after receiving the shock, the total time spent in the dark compartment by the animals of the tramadol-crocin and crocin groups were lower than the tramadol group (*P < 0.05* - *P < 0.01*). But there was no significant difference in the total time spent in the dark compartment in crocin and tramadol-crocin groups (Fig. 3b).

Also 3, 24 and 48 h after receiving the shock, the number of entries to the dark compartment in the tramadol group was significantly higher than the control and saline groups (*P < 0.01*). The results showed that 3, 24 and 48 h after receiving the shock, there was not difference in the number of entries to the dark compartment between the animals of tramadol-crocin and crocin groups with tramadol group (Fig. 3c).

In the tramadol group, the total time spent in the light compartment was lower than the animals of the control and saline groups 3, 24 and 48 h after receiving the shock (*P* < 0.001). The results also showed that 3, 24 and 48 h after receiving the shock, the total time spent in the light compartment by the animals of the tramadol-crocin and crocin groups was higher than that of the tramadol group (*P < 0.05* - *P < 0.01*). Figure 3(d) shows that there was no significant difference in the total time spent in the light compartment between the crocin group in comparison with tramadol-crocin group (Fig. 3d).

### Histopathological data

#### Number of dark neurons in the hippocampus

**Fig. 4:**
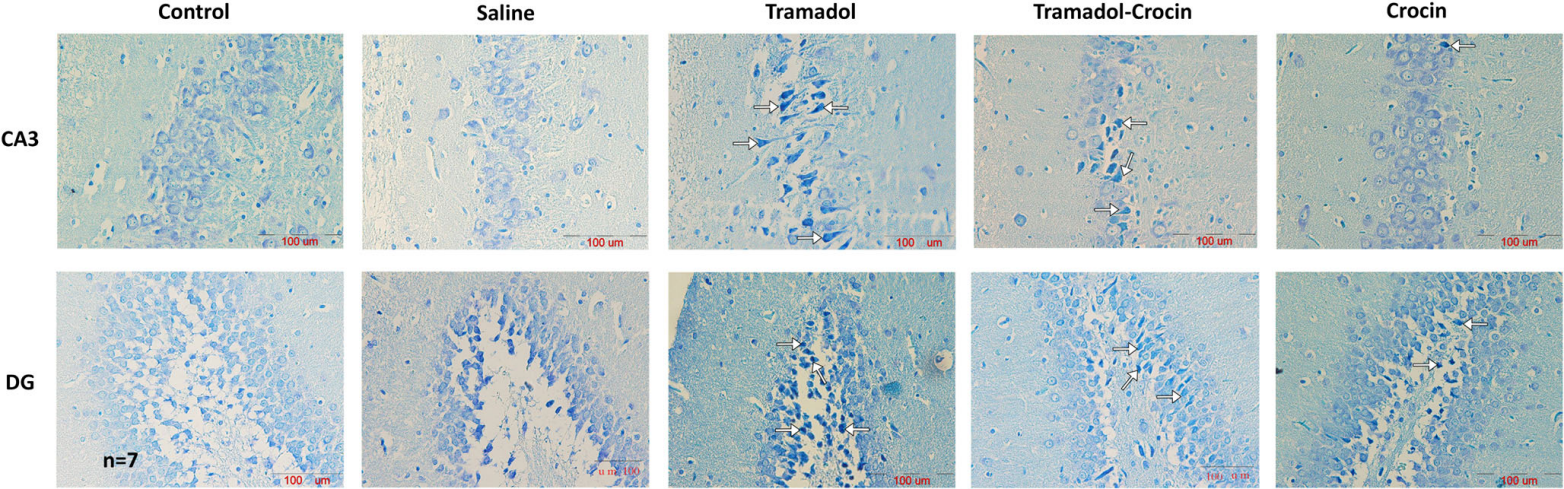
Photomicrographs of the adult rat’s hippocampus in the CA3 and DG. Administration of tramadol to adult rats increased formation of DNs in the hippocampus, while concurrent intake of crocin reduced formation of DNs

**Fig. 5:**
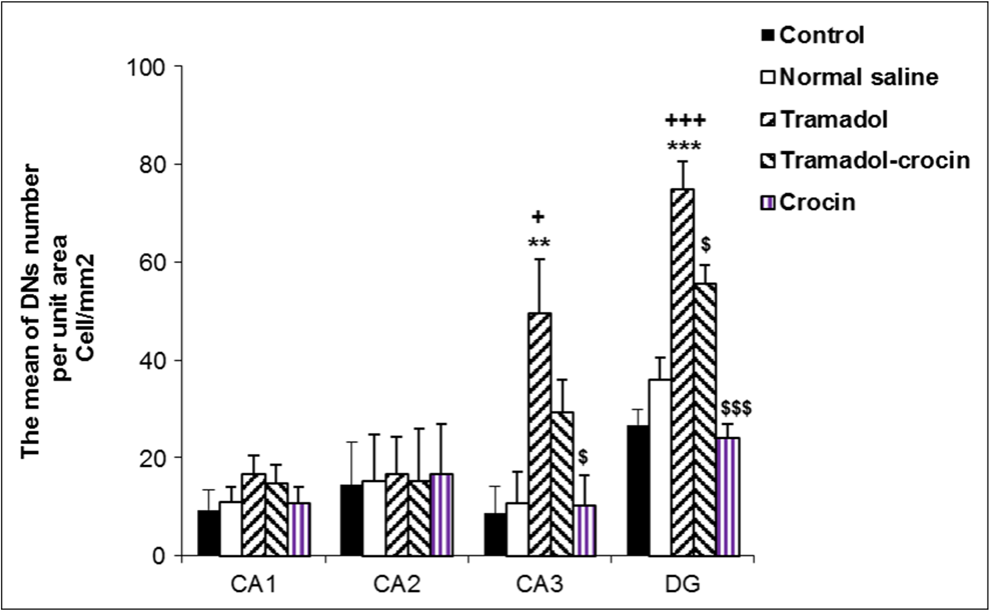
Comparing of DNs mean per unit area (NA) in the CA1, CA2, CA3 and DG of the hippocampus between the five groups using one way ANOVA. Data are expressed as mean ± SEM (*n = 7* in each group). ^**^ *P < 0.01*, ^***^ *P < 0.001* Tramadol group compared with Control group, ^+^ *P < 0.05*, ^+++^ *P < 0.001* Tramadol group compared with Saline group, ^$^ *P < 0.05*, ^$$$^ *P < 0.001* Crocin and Tramadol-Crocin groups compared with Tramadol group

The numbers of DNs per unit area (NA) in CA1, CA2, CA3 and DG of the hippocampus area were counted (Fig. 4). The results showed that there was no significant difference in the numbers of DNs in CA1 and CA2 of hippocampus between the groups. The results also showed that the DNs numbers in CA3 and DG of hippocampus in tramadol group was significantly higher than the control and saline groups (*P < 0.05* - *P < 0.001*). In tramadol-crocin group, the mean of DNs numbers per unit area in DG of hippocampus was lower than the tramadol group (*P < 0.05*). Additionally, in the crocin group, the mean of DNs numbers per unit area in CA3 and DG of hippocampus is less in comparison to the tramadol group (*P < 0.05* - *P < 0.001*) (Fig. 5).

#### Number of apoptotic cells in the hippocampus

**Fig. 6:**
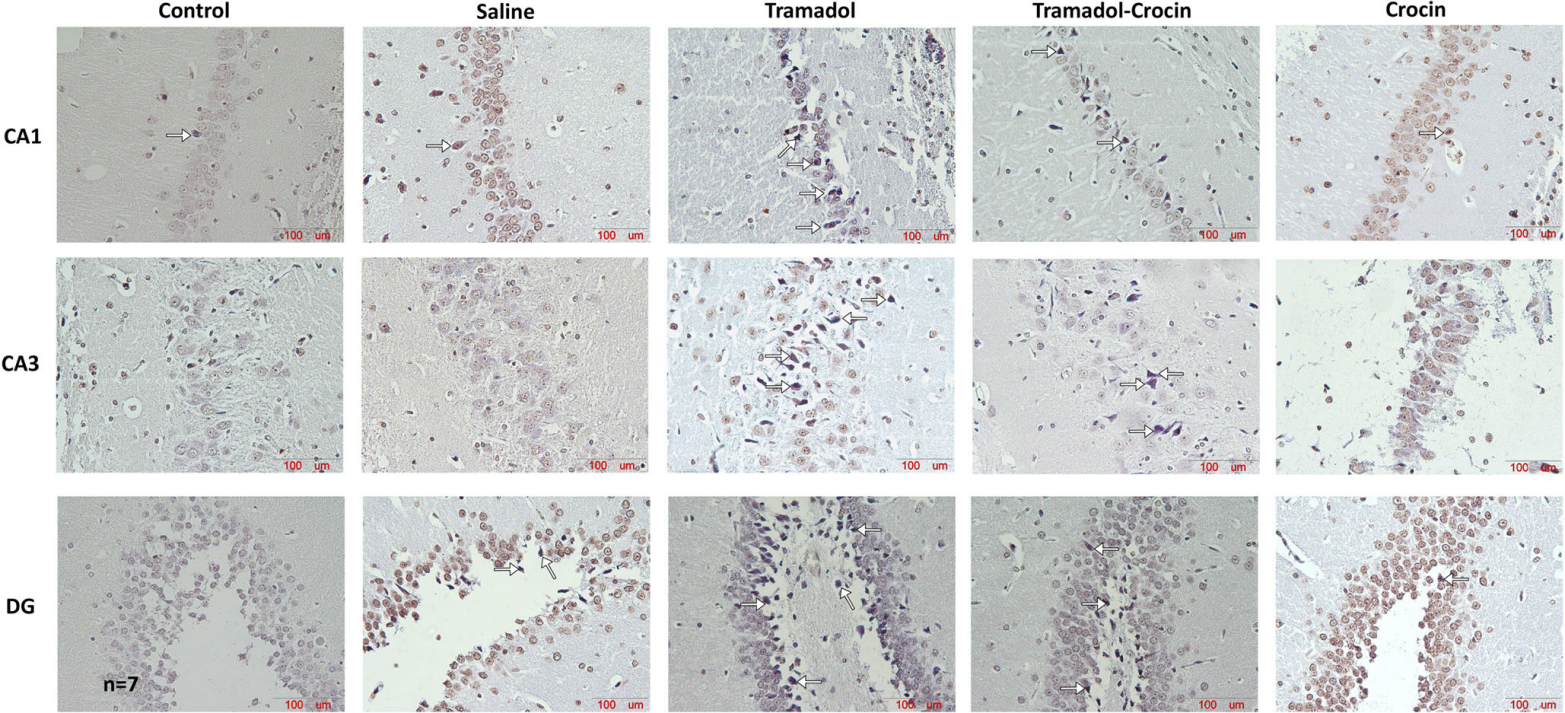
Photomicrographs of the adult rat’s hippocampus in the CA1, CA3, and DG. Administration of tramadol to adult rats increased TUNEL positive cells in the hippocampus, while concurrent intake of crocin reduced TUNEL positive cells

**Fig. 7:**
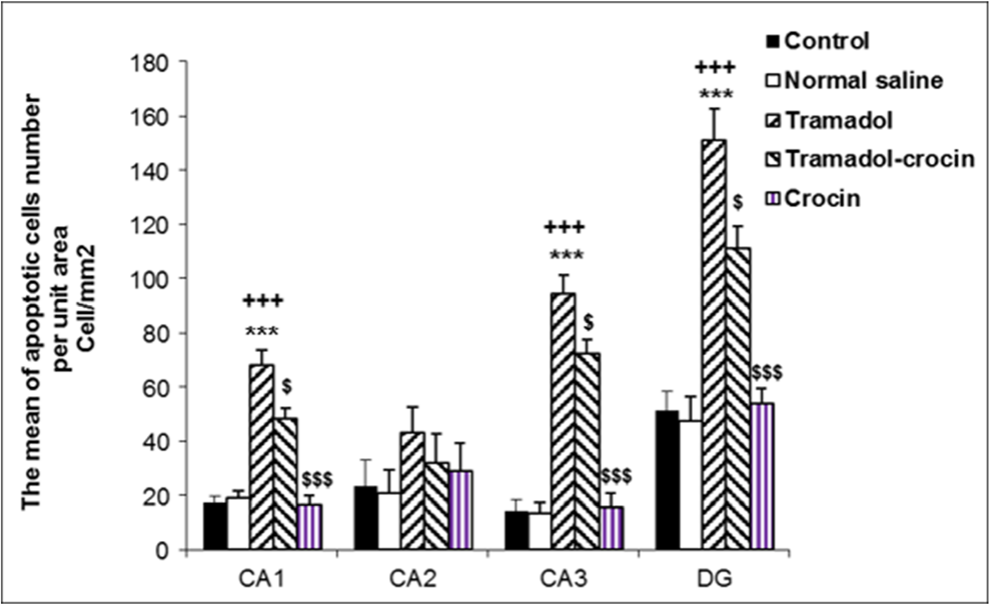
Comparing of TUNEL positive cells mean per unit area (NA) of the CA1, CA2, CA3 and DG of the hippocampus between the five groups using one way ANOVA. Data are expressed as mean ± SEM (*n = 7* in each group). ^***^ *P < 0.001* Tramadol group compared with Control group, ^*+++*^ *P < 0.001* Tramadol group compared with Saline group, ^$^ *P < 0.05*, ^$$$^ *P < 0.001* Crocin and Tramadol-Crocin groups compared with Tramadol group

The TUNEL positive cells per unit area (NA) in CA1, CA2, CA3 and DG of the hippocampus area were counted (Fig. 6). The results showed that the mean of TUNEL positive cell number per unit area in CA1, CA3 and DG of hippocampus in tramadol group was higher than the control and saline groups (*P < 0.001*). In comparison to the tramadol group, the numbers of TUNEL positive cells per unit area in CA1, CA3 and DG of hippocampus decreased significantly in tramadol-crocin group (*P < 0.05*). But there was no significant difference in the numbers of TUNEL positive cells in CA2 of hippocampus between the groups. The results also showed that the numbers of TUNEL positive cells in CA1, CA3 and DG of hippocampus in crocin group was lower than the tramadol group (*P < 0.001*) (Fig. 7).

## Discussion

The results of the present study showed that crocin prevented rats from learning and memory impairments because of the neuroprotective effects on the hippocampal tissues after the administration of tramadol.

Today, tramadol is increasingly being used worldwide, since is believed it has fewer side effects than the other opioids (Loughrey et al. 2003; Zhuo et al. 2012). In addition to this, tramadol is absorbed rapidly and almost entirely when it is orally administered (Grond and Sablotzki 2004). It has also been reported that this drug easily crosses the blood-brain barrier and affects the tissue and functions of the central nervous system (CNS) (Bertaina-Anglade et al. 2006; Hosseini-Sharifabad et al. 2016). Consequently, the effects of repeated oral administration of tramadol on hippocampus tissue, learning and memory were examined. In the present study treatment with tramadol resulted in an inability of the rats to remember the location of the hidden platform in probe test of MWM; which was presented by a shorter traveled distance and less time spent in the target quadrant (Q1) in which the platform was previously located. These results might be considered an evidence for memory impairments in rats by tramadol; however, tramadol did not affect the performances of the rats when they were released to reach the platform during 5 days in MWM test. In the current study, the effect of tramadol administration on motor movements of the rats was not investigated. Nevertheless, the results of a recent study have shown that long-term administration of different doses of tramadol did not effect on rats swimming speed during training days of MWM test (Mehdizadeh et al. 2017). By considering this evidence, it seems the effects of tramadol on rats performance (which was seen in this study) is not because of motor impairing effects. However, it needs more precise investigations in future studies. The results of PA test also confirmed deleterious effects of tramadol on memory, which was presented by a shorter delay time to enter the dark compartment in which they had previously received a shock. The rats also spent a longer time in the dark compartment and entered more frequently to the dark, while they spent a shorter time in the light segment of the apparatus. Consistent with our results, Hosseini-Sharifabad et al. also reported both acute and chronic administration of tramadol impaired learning and memory of the rats when they were examined using object recognition test (Hosseini-Sharifabad et al. 2016).

The responsible mechanism(s) for deleterious effect of tramadol on learning and memory has not been fully understood. Meanwhile, it has been suggested that a decreased level of intracellular signaling molecules such as cAMP, cGMP, PKA, PKC may contribute to deleterious effects of tramadol on neuroplasticity, learning and memory (Bernabeu et al. 1996; Hosseini-Sharifabad et al. 2016; Nakamura et al. 2005). Additionally, it has been reported that tramadol inhibits M1 receptor which may be considered as an explanation for its effects on learning and memory (Nakamura et al. 2005). An inhibitory effect on butyrylcholinesterase and paraoxonase 1 activities has also been suggested to have a role in adverse effects of tramadol on the CNS function (Abdel-Salam et al. 2016).

Moreover, a structural change in neurons has been suggested to occur after the chronic effect of tramadol (Ghoneim et al. 2014). The results of present study confirmed that tramadol increased TUNEL positive cells in rat’s hippocampus compared to the control and saline groups. Tramadol administration also increased DNs in the brain. DNs have been frequently considered a marker of neurotoxicity (Jortner 2005). Considering these results, a neurotoxic effect on the rat’s brains due to repeated tramadol oral administration might be suggested.

Recent studies have suggested that this opioid drug is able to disturb the balance of neurotransmitters in the brain as a result of increased level of glutamate release, while it has an inhibitory effect of GABA system (Abdel-Zaher et al. 2011; Hara et al. 2005; Hassanian-Moghaddam et al. 2013; Rehni et al. 2008). Glutamate and GABA neurotransmitters and their receptors have been frequently reported to play a major role in learning and memory and they also have an important role in neuronal death and neurotoxicity (Collinson et al. 2002; Costa et al. 2015; Hiroshi et al. 1997; McEntee and Crook 1993; Zeng et al. 2007). Glutamate acts as a neurotoxic agent which is frequently attributed to over activity of NMDA receptors (Kawasaki et al. 1997; McEntee and Crook 1993). By using an animal model, learning and memory of the mice and also neuroplasticity impairments and neuronal death of the hippocampus were reported because of an increased level of glutamate in the hippocampus (Zeng et al. 2007). A decreased level of GABA receptors has also been suggested to have a role in neuronal death in a brain schema model (Costa et al. 2015).

On the other hand, neurotoxic effects of tramadol have been sometimes attributed to its adverse effects of antioxidants such as glutathione and inhibition of glutathione peroxidase activity. Tramadol administration has also been reported to be followed by an overproduction of nitric oxide and the brain tissues oxidative damage which presented by a high level malondialdehyde (Abdel-Zaher et al. 2011). Oxidative stress is also suggested to have an important role in DNs formation and apoptosis in hippocampus (Bagheri-abassi et al. 2015). Considering these facts, the effects of crocin as a well-known anti-oxidant anent (Tamaddonfard et al. 2013) on learning and memory of tramadol-treated rats were investigated.

The results of the present study showed that the learning and memory of the trmadol-administrated rats were improved by concurrent administration of crocin. The results showed that crocin administration allowed the rats to remember better the platform location in the probe day of MWM, which was presented by a longer traveled distance in the target quadrant (Q1). The use of crocin was not able to change the time spent and the traveled distance to reach the platform during 5 days of learning in MWM test. Therefore, it seems that crocin improved recall ability but not learning procedures in MWM test. In the present study, the results of PA test also confirmed memory improving effects of crocin in tramadol-treated rats which were presented by a longer delay time to enter the dark compartment, while the time spent in the dark decreased. To the best of our knowledge, the effects of crocin on tramadol induced neurotxicity and cognitive impairment have not been investigated; though the beneficial effects of crocin on CNS including learning and memory have been reported. Ghadrdoost et al. confirmed learning and memory improving effects of crocin using MWM test in a chronic stress model of rats (Ghadrdoost et al. 2011). Additionally, the results of Pitsikas et al. study showed that treatment by 50 mg/ kg of crocin increased delay time of the rats to enter the dark compartment and increased the time spent in the light of PA test. However, the administration of 15–30 mg/kg of crocin didn’t change the behaviors of animals (Pitsikas et al. 2008). Considering these results, it seems that the effect of crocin on learning and memory depends on the used dose. The results of PA test showed that the crocin administration increased time spent in the dark compartment and also increased frequencies of the rats to enter the dark at 48-h post-delivery shock. Conversely, crocin decreased the time spent in the light compartment compared to the control group. Considering these results, the side effects of long-term administration of crocin seems to be investigated in future studies. In a study which was done by Akhondzadeh et al., anxiety like behaviors due to saffron administration has been suggested which may elucidate the results of present study (Akhondzadeh et al. 2005).

In the present study, the histological results confirmed positive effects of crocin on the hippocampus of tramadol-treated rats which may elucidate learning and memory improving effects. The results showed that treatment of the rats by crocin, before each administration of tramadol, decreased the DNs in the CA3 and DG and also TUNEL positive cells in CA1, CA3 and DG regions. Neuroprotective effects of saffron and its constituents have been previously reported (Sadeghnia et al. 2013; Tamaddonfard et al. 2013). Similar to the current study, neuroprotective, protective effects against brain tissues oxidative damage, learning and memory improving properties of crocin in streptozotocin-induced diabetic rats have been previously reported (Ahmadi et al. 2017; Tamaddonfard et al. 2013).

The mechanism(s) responsible for the neuroprotective and learning and memory improving effects of crocin was not investigated in the present study. However, protection against brain tissues oxidative damage and its ability to change neurotransmitters levels for example, reduction of glutamate and increasing GABA in the brain have been suggested to elucidate the effects of crocin (Hosseinzadeh et al. 2008; Moghaddam et al. 2016; Sadeghnia et al. 2013; Tamaddonfard et al. 2013). Saffron has been suggested as a source of acetylcholinesterase inhibitors which may elucidate the effects of crocin which were seen in the present study (Geromichalos et al. 2012; Papandreou et al. 2011). The neuroprotective effects of crocin have been sometimes attributed to its ability to attenuate nitric oxide released in the brain tissues (Nam et al. 2010). Considering these reports and anti-apoptotic effects, saffron and crocin have been considered for their abilities to prevent and to treat neurological diseases (Milajerdi et al. 2015).

## Conclusion

It is concluded that administration of tramadol may disturb learning and memory and it has neurotoxicity effects on inducing DNs formation and apoptosis in rat hippocampus. In addition, crocin may improve/prevent learning and memory disturbances, as well as reduction of DNs and apoptosis in the in tramadol-treated hippocampus of rats.
